# Prostate-specific antigen nadir within 1 year of radiotherapy combined with hormone therapy predicts cancer-specific mortality and biochemical recurrence-free survival in prostate cancer patients

**DOI:** 10.1186/s12894-022-01125-1

**Published:** 2022-11-15

**Authors:** Ilknur Alsan Cetin, Sıtkı Utku Akay, Meric Sengoz

**Affiliations:** 1 Faculty of Medicine, Department of Radiation Oncology, Marmara University, Fevzi Cakmak Mah., Muhsin Yazıcıoglu Cd. No:10, 34899 Pendik, İstanbul, Turkey; 2 Department of Radiation Oncology, Acıbadem University, İstanbul, Turkey

**Keywords:** Prostate cancer, External beam radiotherapy, Hormone therapy, Clinical outcomes

## Abstract

**Background:**

In this study, we investigated the ability of prostate-specific antigen (PSA) 12 months after (nPSA12) external beam radiotherapy (EBRT) combined with androgen deprivation therapy (ADT) to predict biochemical recurrence-free survival (BRFS), overall survival (OS), and prostate cancer-specific mortality (PCSM) in intermediate- and high-risk prostate cancer patients.

**Methods:**

We retrospectively reviewed the clinical data of 338 intermediate- and high-risk prostate cancer patients treated with EBRT with ADT at our institution between 2000 and 2018. The median radiation dose was 76 Gy, the median initial PSA level was 17 ng/mL (range, 1–228 ng/mL), and the median duration of ADT was 24 months (range, 6–167 months). The median PSA level 1 months after EBRT was 0.06 ng/mL (range, 0–25.6 ng/mL). Univariate and multivariate analyses were performed. Patient survival was assessed using the Kaplan-Meier method and Cox proportional hazards regression analyses.

**Results:**

The median follow-up time was 5 years (range, 1–20 years). Multivariate analysis revealed that nPSA was an independent and significant factor associated with OS, PCSM, and BRFS (*P* = 0.008, *P* = 0.001, *P* = 0.04). Furthermore, the time to nPSA12 was an independent predictor of PCSM and BRFS (*P* = 0.042, *P* = 0.021). Pelvic irradiation was also significantly associated with worse OS and PCSM (*P* = 0.004, *P* = 0.01). Additionally, age (≤ 70 or > 70 years) and hormone therapy duration (6 months, 1–3 years, or > 3 years) were significantly associated with OS and PCSM, respectively (*P* = 0.004, *P* = 0.02). For high risk, nPSA and nPSA12 were an independent predictor for BRFS. (*P* = 0.021, *P* = 0.029)

**Conclusion:**

The nPSA12 level of > 0.06 ng/mL may independently predict worse PCSM and BRFS in intermediate- and high-risk prostate cancer patients undergoing EBRT and ADT. Additionally, for high risk, nPSA > 0.06 ng/mL and nPSA12 > 0.06 ng/mL may independently predict worse BRFS.

## Background

According to National Comprehensive Cancer Network (NCCN) clinical practice guidelines, patients with localized prostate cancer can be classified based on their clinical outcomes as low-, intermediate-, and high-risk [1, 2]. Intermediate- and high-risk patients with localized prostate cancer are often treated with definitive external beam radiation therapy (EBRT) combined with androgen deprivation therapy (ADT). Numerous large-cohort phase III trials have demonstrated that the combination of ADT and EBRT can significantly improve prostate cancer-specific mortality (PCSM), distant metastasis (DM), and biochemical recurrence (BR) rates [3–7].

The measurement of serum prostate-specific antigen (PSA) levels is an invaluable biochemical method for prostate cancer screening, treatment response monitoring, and disease recurrence detection. The nadir in prostate-specific antigen (nPSA) after radiotherapy (RT) has been shown to predict BR, DM, cause-specific mortality (CSM), and overall mortality (OM) [8–13]. Additionally, mounting evidence suggests that time-limited measures of PSA are independent early predictors of BR and DM in patients undergoing definitive EBRT [14–16]. However, the prognostic value of nPSA in prostate cancer patients treated with concurrent ADT and EBRT remains unclear.

The aim of this study was to determine whether a 12-month post-treatment nPSA (nPSA12) cutoff value of 0.06 ng/mL can serve as an early predictor of biochemical recurrence-free survival (BRFS), PCSM, and overall survival (OS) in prostate cancer patients treated with concurrent ADT and EBRT.

## Methods

We retrospectively reviewed the clinical data of 338 intermediate- and high-risk prostate cancer patients who underwent radiotherapy combined with ADT between 2000 and 2018 at our institution. All patients had biopsy-confirmed adenocarcinoma of the prostate and underwent serum PSA testing before treatment. The T stage was determined based on a digital rectal examination. Based on NCCN guidelines, patient risk was classified as intermediate or high. Patient characteristics, including the clinical stage, are shown in Table 1.

**Table 1:** Patient characteristics, treatment, and univariate analysis results

	Number of patients (N = 338) (%)	OS	PCSM	BRFS
		**P value**	**P value**	**P value**
**Age**		**0.003**	0.4	0.16
Median	71(50–85)			
≤70	157(46.4)			
>70	181(53.6)			
**Comorbidity**		0.5	0.33	**0.023**
no	120(35.7)			
yes	218(64.3)			
**% Positive biopsies**		0.9	**0.01**	0.3
< 0.5	154(45.6)			
≥ 0.5	184(54.4)			
**% Tumor volume**		0.8	**0.006**	0.07
< 50%	150(44.4)			
≥ 50%	188(55.6)			
**Gleason score**		0.27	**0.03**	**0.004**
**< 7**	90(26.6)			
**7**	154(45.6)			
**> 7**	94(27.8)			
**Histological grade group**		0.31	0.1	**< 0.0001**
1 (≤ 6 GS)	91(26.9)			
2 (3 + 4 GS)	95(28.1)			
3 (4 + 3 GS)	58(17.2)			
4 (8 GS)	48(14.2)			
	46(13.6)			
**Risk Group**		0.69	**0.045**	**0.005**
intermediate	131(38.8)			
high	207(61.2)			
**Initial PSA ng/ml**		0.38	0.44	**0.034**
<10	75(30)			
10–20	110(32.5)			
**>**20	153(45.5)			
**Clinical stage**		0.6	0.09	0.9
T1	49(14.5)			
T2	251(74.3)			
T3	34(10.2)			
T4	4(1)			
**HT use**		**0.047**	0.3	0.7
Neoadjuvant HT	271(80)			
concomitant	59(17.5)			
Adjuvant	8(2.5)			
**HT period**		0.17	**0.001**	0.09
6 month	89(26.3)			
1–3 year	206(60.9)			
> 3 year	43(12.7)			
**RT dose**		0.68	0.26	**0.001**
70 Gy	56(16.6)			
70-76 Gy	205(60.7)			
>76	77(22.8)			
**RT field**		**0.006**	**< 0.0001**	**< 0.0001**
Prostate	265(78.4)			
Pelvis	73(21.6)			
**RT Technic**		**0.1**	0.14	**< 0.0001**
3DCRT	208(61.5)			
IMRT(VMAT)	130(38.5)			
**PSA nadir (ng/mL)**		**0.002**	**< 0.0001**	**< 0.0001**
≤ 0.06	269(79.6)			
> 0.06	56(16.6)			
Unknown	13(3.8)			
**Time to nadir PSA**		0.72	**0.017**	**0.003**
< 12 month	234(69.2)			
≥ 12 month	92(26.9)			
unknown	13(3.8)			

BOS: Overall survival, PCSM: cancer-specific survival, RFS: biochemical recurrence-free survival, PSA:prostate-specific antigen, HT: hormonotherapy, RT: radiotherapy, 3DCRT: three-dimensional conformal radiotherapy, IMRT: Intensity modulated radiotherapy, VMAT: Volumetric Modulated Arc Therapy

All patients were treated with either high-dose 3-dimensional conformal radiation therapy (3DCRT) or volumetric-modulated arc therapy, delivering 70–78 Gy in 28–39 fractions. For intermediate-risk patients, the clinical target volume (CTV) included in the prostate and the seminal vesicles, and 54 Gy was delivered in the first phase of treatment. In the second phase, the dose delivered to the prostate was increased to 70–76 Gy. For high-risk patients, the CTV included the prostate, seminal vesicles, and pelvic lymph nodes; 54 Gy delivered in the first phase of treatment. In the second phase, the dose was increased to 74–78 Gy. The risk of lymph node involvement was calculated according to the Roach formula, and patients with a high risk (> 15%) of lymph node metastasis received pelvic radiation. The dose was typically prescribed at the 95% isodose of the beam arrangement and was normalized so that the planning treatment volume was included within the 95% isodose line. All patients were treated with 6 MV photons and received neoadjuvant or concurrent ADT. All patients were also treated with luteinizing hormone-releasing hormone agonists (Goserelin 10.8 mg/3 months, leuprolide acetate 11.25 mg/3 months or 22.5 mg/3months) plus nonsteroidal antiandrogens (bicalutamide 50 mg once daily, orally administered for 1 month). Typically, intermediate-risk patients underwent ADT for 6 months, whereas high-risk patients received ADT for 1–3 years. Neoadjuvant ADT was preferred in patients with large prostate tumors.

The treatment response was monitored by serum PSA testing every 3 months during ADT. Patients had the PSA value of at least 4 after treatment. Patients were followed-up every 3 months for the first 2 years, every 6 months for the following 3 years, and annually thereafter. We defined nPSA as the lowest PSA achieved after treatment. Time to nPSA was defined as the time from treatment initiation to nPSA achievement. BR was defined as a PSA level of at least 2 ng/mL above the nPSA, as per the Phoenix BR definition. Tumor metastases were detected radiographically, and the cause of death was recorded. We collected at least 4 post-treatment PSA values for each patient. nPSA12 was defined as a PSA level of 0.06 ng/mL achieved in the first year of radiotherapy completion. All patients were followed-up for at least 1 year.

BRFS, PCSM, cancer-specific survival (CSS), and OS were determined from the date of RT completion, and patients were stratified based on nPSA12 levels (≤ 0.06 ng/mL or > 0.06 ng/mL). Survival time was defined as the time between the last radiotherapy session and the last follow-up or death. OS was defined as the time between disease diagnosis and the follow-up or death. Patient survival was analyzed using the Kaplan‑Meier method and the log‑rank test. Univariable and multivariable Cox regression analyses were performed to assess the prognostic value of nPSA12 levels. Hazard ratios (HRs) and 95% confidence intervals (CIs) were calculated. All statistical analyses were conducted using SPSS version 22.0 (IBM Corp., Armonk, NY). *P*-values < 0.05 were considered statistically significant.

## Results

**Fig. 1:**
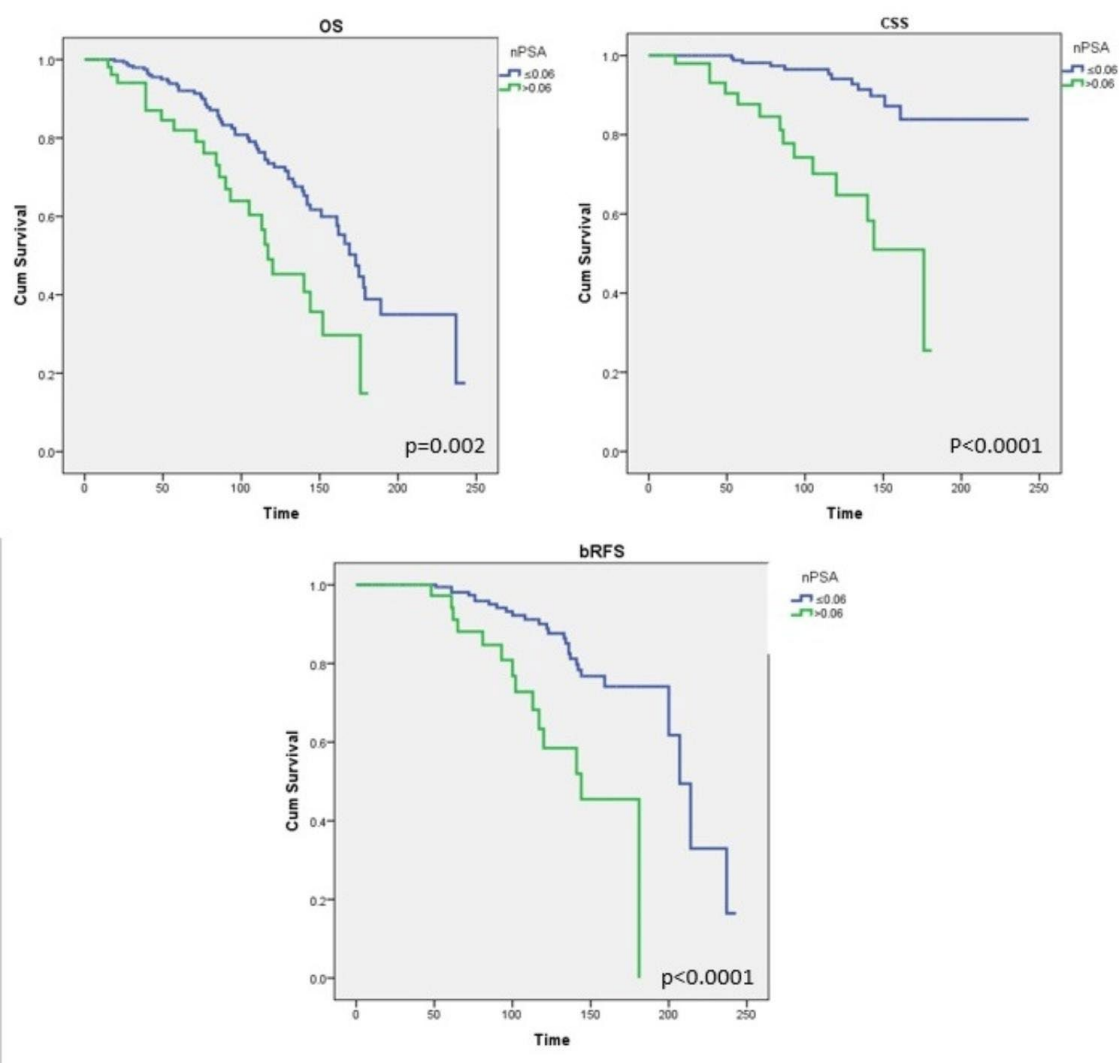
Univariate analysis assessing the ability of nPSA levels (≤ 0.06 ng/mL or > 0.06 ng/mL) to predict OS, PCSM, and BRFS.

The median follow-up time after RT completion was 5 years (range, 1–20 years). Univariate analyses revealed that advanced age (> 70 years) (SE = 11.4, 95%CI = 139.5-184.4; *P* = 0.003), concurrent hormone therapy (SE = 11.4, 95%CI = 139.5-184.4, *P* = 0.04), pelvic irradiation (SE = 11.4, 95%CI = 139.5-184.4, *P* = 0.006), and nPSA > 0.06 ng/mL (SE = 9.8, 95%CI = 146.7-185.2, *P* = 0.002) were significantly associated with poor OS. Positive biopsy (≥ 0.5%) (SE = 5.9, 95%CI = 199.8-223.3, *P* = 0.01), tumor size (≥ 50%) (SE = 5.9, 95%CI = 201.6-224.7, *P* = 0.006), Gleason score (> 7) (SE = 5.8, 95%CI = 200.6-223.4, *P* = 0.03), high risk (SE = 5.8, 95%CI = 200.6-223.4, *P* = 0.045), prolonged hormone therapy (> 3 years) (SE = 5.8, 95%CI = 200.6-223.4, *P* = 0.001), pelvic irradiation (SE = 13, 95%CI = 79.2-130.7, *P* < 0.0001), nPSA > 0.06 ng/mL (SE = 20.7, 95%CI = 135.3-216.6, *P* < 0.0001), and prolonged time to nPSA (≥ 12 months) (SE = 5.9, 95%CI = 199.5-222.9, *P* = 0.017) were significantly associated with poor CSS (Fig. 1).

**Fig. 2:**
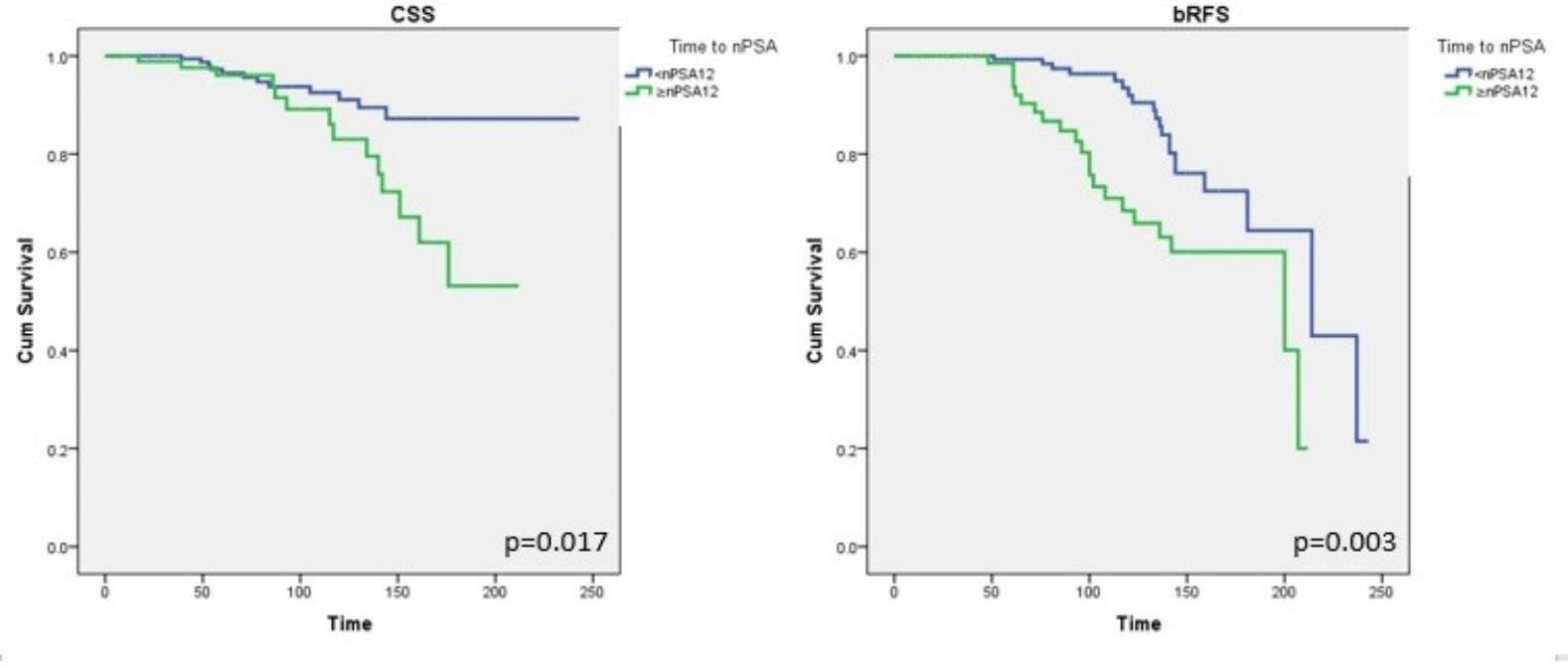
Univariate analysis assessing the ability of nPSA12 to predict PCSM and CSS.

Comorbidities (SE = 14.8, 95%CI = 177.8-236.1, *P* = 0.023), Gleason score (> 7) (SE = 15.9, 95%CI = 175.7-238.2, *P* = 0.004), histological grade (grades 4 and 5) (SE = 15.9, 95%CI = 175.7-238.2, *P* < 0.0001), high risk (SE = 15.9, 95%CI = 175.7-238.2, *P* = 0.005), initial PSA levels (> 20 ng/mL) (SE = 15.9, 95%CI = 175.7-238.2, *P* = 0.034), RT dose (> 76 Gy) (SE = 15.9, 95%CI = 175.7-238.2, *P* = 0.001), pelvic irradiation (SE = 15.9, 95%CI = 175.7-238.2, *P* < 0.0001), IMRT technique (SE = 15.9, 95%CI = 175.7-238.2, *P* < 0.0001), nPSA > 0.06 ng/mL (SE = 15.9, 95%CI = 175.6-238.3, *P* < 0.0001), and prolonged time to nPSA (≥ 12 months) (SE = 15.9, 95%CI = 175.6-238.3, *P* = 0.003) were significantly worse associated with BRFS (Table 1; Fig. 2).

Multivariate analyses revealed that nPSA ≤ 0.06 ng/mL was independently and significantly better associated with OS (*P* = 0.008), PCSM (*P* = 0.001), and BRFS (*P* = 0.04). Similarly, time to nPSA < 12 month was independently and significantly better associated with PCSM (*P* = 0.042) and BRFS (*P* = 0.021). RT small (prostate) field prostate was significantly better associated with OS (*P* = 0.004) and PCSM (*P* = 0.01). Furthermore, age ≤ 70 was independently associated with OS (*P* = 0.004) and hormone therapy period (6 months) was independently better associated with PCSM (*P* = 0.02; Table 2).

**Table 2:** Multivariate analysis results showing the prognostic value of different clinicopathological parameters

	OS		PCSM		BRFS	
	HR	p	HR	p	HR	p
**Age**	1.9(1.2–3.1)	**0.004**				
**HT period**			2.8(1.1–6.9)	**0.02**		
**RT field**	2.1(1.1–3.9)	**0.015**	3.8(1.3–10)	**0.01**	0.5(1.6–1.8)	0.3
**RT Technic**			0.5(0.1–1.9)	0.35	0.6(0.2–1.9)	**0.45**
**PSA nadir**	1.9(1.1–3.1)	**0.008**	3.9(1.7–9.3)	**0.001**	2(0.9–4.1)	**0.04**
**Time to nadir PSA**	0.9(0.6–1.6)	0.952	2.4(1-5.6)	**0.042**	2.1(1.1-4)	**0.021**

OS: Overall survival, PCSM; prostate cancer specific mortality, BRFS: biochemical recurrence-free survival, HT: hormonotherapy, RT: radiotherapy, HR: hazard ratio

In univariate analysis and multivariate analysis, intermediate and high risk PC were analyzed separately. For intermediate risk, statistically significant difference was found in nPSA for OS, PCSM, BRFS (*P* = 0.027, *P* < 0.0001, *P* = 0.041). In addition, statistically significant difference was found for PCSM in % positive biopsies, % tumor volume and nPSA12 (*P* = 0.028, *P* = 0.003, *P* = 0.021). For high risk, statistically significant difference was found in nPSA for OS, PCSM, BRFS (*P* = 0.034, *P* < 0.0001, *P* = 0.005). In addition, statistically significant difference was found for OS in age (*P* = 0.02) and for BRFS in RT dose (*P* = 0.037), nPSA12 (*P* = 0.03). nPSA (≤ 0.06 ng/mL )(*P* = 0.021) and nPSA12 (time to nPSA < 12 month) (*P* = 0.029) were found to be independent and significantly better predictive factors for BRFS in multivariate analysis.

The 10- and 15-year cumulative incidences of PCSM were 88.8% and 74%, respectively. The 10- and 15-year cumulative incidences of BRFS were 84.3% and 69.4%, respectively. For nPSA12 ≤ 0.06 ng/mL, the 10- and 15-yearcumulative incidences of PCSM and BRFS were 96.5%, 87.2% and 99.3%, 72.5% respectively. Grades 1 and 2 rectal toxicity were observed in 80 (23%) and 18 (5%) patients. Moreover, grades 1 and 2 late urinary toxicity were observed in 180 (53%) and 20 (6%) patients. No cases of severe toxicity (grades 3 and 4) were observed. Erectile function was affected in 113 (33%) patients.

## Discussion

In this study, we found that nPSA levels of > 0.06 ng/mL within 12 months after ADT and EBRT predicted worse clinical outcomes in intermediate- and high-risk prostate cancer patients. Importantly, nPSA12 > 0.06 ng/mL was significantly associated with poor PCSM, and BRFS. Similarly, pelvic irradiation, > 70 years of age, and prolonged (> 3 years) hormone therapy were associated with poor OS and PCSM.

The relationship between BRFS and different nPSA levels has been previously investigated [2, 8, 17]. These previous studies have shown that the combination of antiandrogens with RT decreased PSA levels and that ADT led to lower nPSA values [18, 19]. Patel et al. [20] reported a strong association between an early post-RT PSA level of ≥ 0.09 ng/mL and high risks of BF, DM, PCSM, and death from all causes. Geara et al. [21] found that an nPSA level of 0.06 ng/mL was an independent predictor of BFS in patients with intermediate- or high-risk prostate cancer undergoing definitive EBRT and ADT. Consistently, we found that nPSA levels > 0.06 ng/mL were associated with worse OS, PCSM, and BRFS.

Cavanaugh et al. [22] demonstrated a strong association between PSA levels and BR, CSM, and OM. Alcantara et al. [15] identified nPSA12 (≤ 2 versus > 2 ng/mL) as an early predictor of BF, DM, and mortality, independent of RT dose and outcome determinants after RT. 10-year DM rates for nPSA12 ≤ 2 versus > 2 ng/mL were 4% versus 19% (P < 0.0001). Ray et al. [14] a total of 4839 patients were treated for Stage T1-T2 prostate cancer with RT and without hormone therapy. 8-year rate of PSA-DFS, DMFS, and OS in patients with a trough PSA12 (≤ 2.0 ng/mL or > 2 ng/mL) 55%, 95% and 73% respectively or 40%, 88% and 69% respectively. In the study of Ogawa et al. [23] 84 patients with localized prostate cancer were treated with RT and hormone therapy. 3-year PFS rate in patients with nPSA12 levels < 0.5 ng/mL and patients with nPSA12 levels ≥ 0.5 ng/mL were 96% and 44%, respectively (p < 0.0001). In this study, for nPSA12 (≤ 0.06 ng/mL or > 0.06 ng/mL), the 8 year incidences of PCSM and BRFS were 91.1%, 92% respectively or 83.2%, 65.9% respectively. We found that nPSA12 was an independent predictor for PCMS, BRFS in intermediate- and high-risk prostate cancer patients after ADT and EBRT.

The clinical usefulness of pelvic irradiation in patients with a high risk of lymph node metastasis remains controversial. In this study, we found that pelvic irradiation worsened patient survival. Patients with high risk and receiving pelvic RT may have poorer survival as they have a more locally advanced stage. For this reason, PCSM at intermediate risk prostate cancer patients was 94.6%-81.1% at 10–15 years, while it was 83.8%-70.8% at high risk prostate cancer patients (p = 0.04). In addition, Prostate cancer is more common among the elderly, especially in older men with comorbidities. Here, we found that comorbidities were the cause of approximately 70% of all deaths.

Numerous randomized studies have shown that the combination of prolonged hormone therapy (1–3 years) with RT may benefit high risk prostate cancer patients. In this study, we found that hormone therapy for more than 3 years led to worse PCSM. This can be explained by the fact that these patients are very high risk patients.

## Conclusion

In conclusion, our findings suggest that nPSA12 levels of > 0.06 ng/mL within 12 months after ADT and EBRT may be a useful prognostic factor of worse survival outcomes in intermediate- and high-risk prostate cancer patients.

## Data Availability

The datasets used and/or analyzed during the current study are available from the corresponding author on reasonable request.

## List of abbreviations

3DCRT: 3-dimensional conformal radiation therapy
ADT: Androgen deprivation therapy
BR: Biochemical recurrence
BRFS: Biochemical recurrence-free survival
CI: Confidence intervals
CTV: Clinical target volume
CSM: Cause-specific mortality
CSS: Cancer-specific survival
DM: Distant metastasis
EBRT: External beam radiotherapy
IMRT: Intensity Modulated Radiotherapy
HR: Hazard ratios
nPSA12: 12 months after PSA
NCCN: National Comprehensive Cancer Network
OM: Overall mortality
OS: Overall survival
PCSM: Prostate cancer-specific mortality
PSA: Prostate-specific antigen
RT: Radiotherapy
SE: Standard error
